# Exposure to Organochlorine Pollutants and Type 2 Diabetes: A Systematic Review and Meta-Analysis

**DOI:** 10.1371/journal.pone.0085556

**Published:** 2014-10-15

**Authors:** Mengling Tang, Kun Chen, Fangxing Yang, Weiping Liu

**Affiliations:** 1 MOE Key Laboratory of Environmental Remediation and Ecosystem Health, College of Environmental and Resource Sciences, Zhejiang University, Hangzhou, China; 2 Department of Epidemiology & Health Statistics, School of Public Health, Zhejiang University, Hangzhou, China; University of Tolima, Colombia

## Abstract

**Objective:**

Though exposure to organochlorine pollutants (OCPs) is considered a risk factor for type 2 diabetes (T2DM), epidemiological evidence for the association remains controversial. A systematic review and meta-analysis was applied to quantitatively evaluate the association between exposure to OCPs and incidence of T2DM and pool the inconsistent evidence.

**Design and Methods:**

Publications in English were searched in MEDLINE and WEB OF SCIENCE databases and related reference lists up to August 2013. Quantitative estimates and information regarding study characteristics were extracted from 23 original studies. Quality assessments of external validity, bias, exposure measurement and confounding were performed, and subgroup analyses were conducted to examine the heterogeneity sources.

**Results:**

We retrieved 23 eligible articles to conduct this meta-analysis. OR (odds ratio) or RR (risk ratio) estimates in each subgroup were discussed, and the strong associations were observed in PCB-153 (OR, 1.52; 95% CI, 1.19–1.94), PCBs (OR, 2.14; 95% CI, 1.53–2.99), and *p,p′*-DDE (OR, 1.33; 95% CI, 1.15–1.54) based on a random-effects model.

**Conclusions:**

This meta-analysis provides quantitative evidence supporting the conclusion that exposure to organochlorine pollutants is associated with an increased risk of incidence of T2DM.

## Introduction

Organochlorine pollutants (OCPs), represented by DDT (Dichlorodiphenyltrichloroethane) and PCBs (Polychlorinated biphenyls), are environmental contaminates of global concern because of their potential for bio-accumulate and bio-magnify in ecosystems and hazardous effects on human health. Though DDT and PCBs were forbidden in most countries in the 1970s [1] and 1980s [2], and the concentrations of these chemicals in the environment, organisms and human tissues were decreasing over the past 30 years, they can still be detected due to their characteristics of persistency, semi-volatility, lipid solubility, bioaccumulation and biomagnification [1]. In Ghana, DDE (Dichlorodiphenyldichloroethylene) was detected at the highest levels among DDT isomers at 44.8 and 7.1 ng/g in breast milk and serum, respectively [3]. In China, PCBs were detected at 0.9 ng/g in lipid in the placentas of women who had pregnancies affected by neural tube defects and at levels of 0.87 ng/g in lipid controls [4].

Type 2 diabetes mellitus (T2DM), formerly called adult-onset diabetes, is a noninsulin-dependent diabetes that accounts for 90–95% of all diabetes cases [5]. As a result of a metabolic disorder of glucose, T2DM has become a major global epidemic in recent years, and its prevalence will likely double over the next 20 years [6]. World Health Organization (WHO) projects that diabetes will be the 7^th^ leading cause of death in 2030. The prevalence of T2DM may be affected by the interaction of conventional risk factors and a combination of genetic susceptibility [7], metabolic syndromes such as obesity [8] and hypertension [9], age, race, and poor diet. In addition, the accumulation of environmental pollutants in the human body has been suggested to have a significant contribution to the disease [10].

Within different populations, the positive associations were observed in the epidemiological studies about T2DM risk exposure to OCPs [11], [12]. The associations may be attributed to certain mechanisms of the active ingredients of OCPs, such as γ-aminobutyric acid, which affect the neurotransmitter or ion channel systems involved in regulating pancreatic function and then influence glucose homeostasis [13]. Toxic effects through direct binding and activation of the aryl hydrocarbon receptor (AhR) pathway [14] and mediation through AhR-independent oxidative stress and mitochondrial dysfunction [15] have also been reported as biological mechanisms. Furthermore, toxic effects on estrogen receptor, peroxisome proliferator-activated receptor γ (PPARγ), and progesterone receptor were considered other mechanisms. However, the pathogenesis of exposure to OCPs is currently obscure.

To our knowledge, there have been many epidemiological studies regarding the association between exposure to OCPs and the prevalence of T2DM. However, the results showed contradictory. In order to fully evaluate and characterize the association and fill the vacancy of epidemiological evidence in the comprehensive summary, we performed a sub-group meta-analysis of the results of T2DM risk from exposure to OCPs. We systematically analyzed all studies on T2DM risk from exposure to OCPs up to August 2013.

## Methods

### Study Identification

We reported the meta-analysis according to the Preferred Reporting Items for Systematic Reviews and Meta-analyses (PRISMA) [16] (Checklist S1). Publications about epidemiological evidence of T2DM risk from exposure to OCPs were identified by a search on MEDLINE (National Library of Medicine, Bethesda, MD) and WEB OF SCIENCE databases. A preliminary total of 116 related studies published up to August 2013 were selected using various combinations of the following keywords: “diabetes”, “DDE”, “DDT”, “PCB”, and “organochlorine” with no restriction of publication type and date. The reference lists of the relevant publications identified were checked for additional studies and the recent articles in relevant journals were also scanned to identify other potential studies. The whole search was limited to studies published in English in the open literature in peer-reviewed journals.

### Criteria for Inclusion

The systematic review and identification of eligible studies was performed. The titles and abstracts were screened to determine their relevance to the diabetes effects of humans when exposed to OCPs. The full text of potentially relevant studies was then examined and the eligibility criteria were applied to select the included studies.

A publication was considered eligible for review if it fulfilled the following six inclusion criteria. (1) It must be an original epidemiologic study using a case-control, cross-sectional, or prospective study design and other types of reviews, meta-analysis, case-reports, comments, letters, editorials, abstracts were excluded. (2) Papers should be written in the English language. (3) OCP exposure levels had to be measured in actual tissue samples (serum or serum lipid), not by environmental data or other indirect ways. (4) It unequivocally reported measures of association, including odds ratios (OR) and relative risk (RR) and confidence intervals (CIs) for diabetes risk and also considered papers that did not report these measures directly, but were able to extrapolate the relevant values. (5) Studies should use biomarkers of OCPs within our selected ones including PCB-153, PCBs, and *p,p′*-DDE, while others OCPs, for instance, using PCB-126 as the biomarker, were not included. (6) In addition, T2DM was confirmed by self-report or hospital diagnosis, and diseases related to T2DM as insulin resistence were excluded. Finally, 23 epidemiological studies were extracted for further systematic analysis.

### Data Extraction

The authors examined the articles and independently extracted and tabulated the information. A standard data abstraction form was created to record the following information for each suitable article: first author name, year of publication, geographic region of the studies, epidemiologic design, subject selection, exposure pathways, type of OCP, biologic specimens, number of cases and controls, and a risk index calculated with the categories of the exposure and referent, corresponding 95% CI for T2DM. The risk indexes, adjusted for different confounding such as sex, BMI, cigarette smoking, and the ones stratified by age and sex were all extracted. The results of this abstraction were compared between the authors and consensus was obtained before the meta-analysis.

Stratification of the data were performed focusing on several variables that could influence the results, including exposure levels (background or high concentration exposure), study design (case-control, perspective, or cross-sectional study), population selection (general population or women), and biologic specimen (serum or serum lipid).

### Quality Assessment

In order to assess evidence, all included studies underwent an independent quality assessment modified from the versions of the 1998 Downs and Black [17] and Wigle et al. checklists [18]. We discussed the individual items on the checklist to clarify their interpretation before conducting the quality assessment. The same to the version made by the latter group, we also added exposure measurement as the internal validity assessment to the checklist of quality assessment. However, some items that were either related only to reporting or were not applicable were removed from the checklist. No attempt was made to blind the reviewers of the authorship or publication status of the original studies. The evaluated factors including the representativeness of the selected participants, bias, and confounding were given a mark to assess the article quality. Finally, a total of 13 items and 16 scores were listed (Table S1). The results with higher scores were considered to be of superior quality. Differences in quality assessment were resolved by consensus.

### Data Analysis

The heterogeneity across individual studies was quantified by the *Q*-test and *I* ^2^-test: when the result of the *Q*-test showed evidence of the heterogeneity (p<0.1), we used the random-effect analysis; otherwise, the *I* ^2^-test, which interpreted *I* ^2^ values of 25%, 50%, 75% as low, moderate, and high degrees of heterogeneity respectively, was used to assess heterogeneity. This is because the *Q*-test has low statistical power with few studies [19] and the fixed-effect analysis was conducted when *I* ^2^<25%. p<0.10 or *I* ^2^>25% was considered significant heterogeneity which questions the validity of pooled estimates. The *I* ^2^ describes the percentage of total variation across studies due to heterogeneity rather than chance [20]. When heterogeneity exists, subgroup analyses were conducted to investigate potential sources of heterogeneity.

The risk estimates of OR or RR were combined for the evaluation of the dose-response relation between OCP exposure and T2DM prevalence. We assumed similarity between the OR and RR. When combined these binary variables, we aimed to choose the ones calculated between the highest exposed group and the references and the ones with the most adjusted variables. We attempted to combine adjusted OR or RR from primary studies, but if not possible, we pooled raw outcome data to yield unadjusted OR. In addition, we combined the risk estimates which were calculated by OCPs concentrations tested in serum lipid, otherwise we choose the values tested directly in serum. We considered all the OR stratified by ages and by BMI in each study. To conduct meta-analyses, we defined the least group as 4 articles with risk estimates [21], which corresponded to a minimum of 100 cases of T2DM.

We performed meta-analyses using Review Manager (RevMan) version 5.0 (Nordic Cochrane Centre, Cochrane Collaboration, Copenhagen, Denmark) to evaluate the overall risk of T2DM caused by exposure to OCPs. For the risk estimates presented as a binary variable, such as OR and RR, the inverse of variance for fixed-effects models using the Mantel-Haenszel [22] method which assumes that results across studies differ only by sampling error. The DerSimonian and Laird method [23] for random-effects models were used to combine the overall binaries and their corresponding 95% CI. The results of meta-analysis including all subgroup analysis were illustrated by forest plots.

Publication bias due to study size was investigated by visual inspection of funnel plots which showed the natural logarithm of the estimate of RR (lnRR) versus the inverse of standard error (1/SE). Funnel plot asymmetry can be illustrated by factors as the non-publication of small studies with negative results, differences in study quality and study heterogeneity.

To determine whether some of the decisions we made had a major impact on the results of the review, sensitivity analyses were conducted by (1) removing studies with the highest and lowest percentage weight in all included studies, (2) deleting studies with highest and lowest quality scores, (3) excluding studies reporting the lowest or highest estimator of binary variables.

## Results

### Study Characteristics

**Figure 1:**
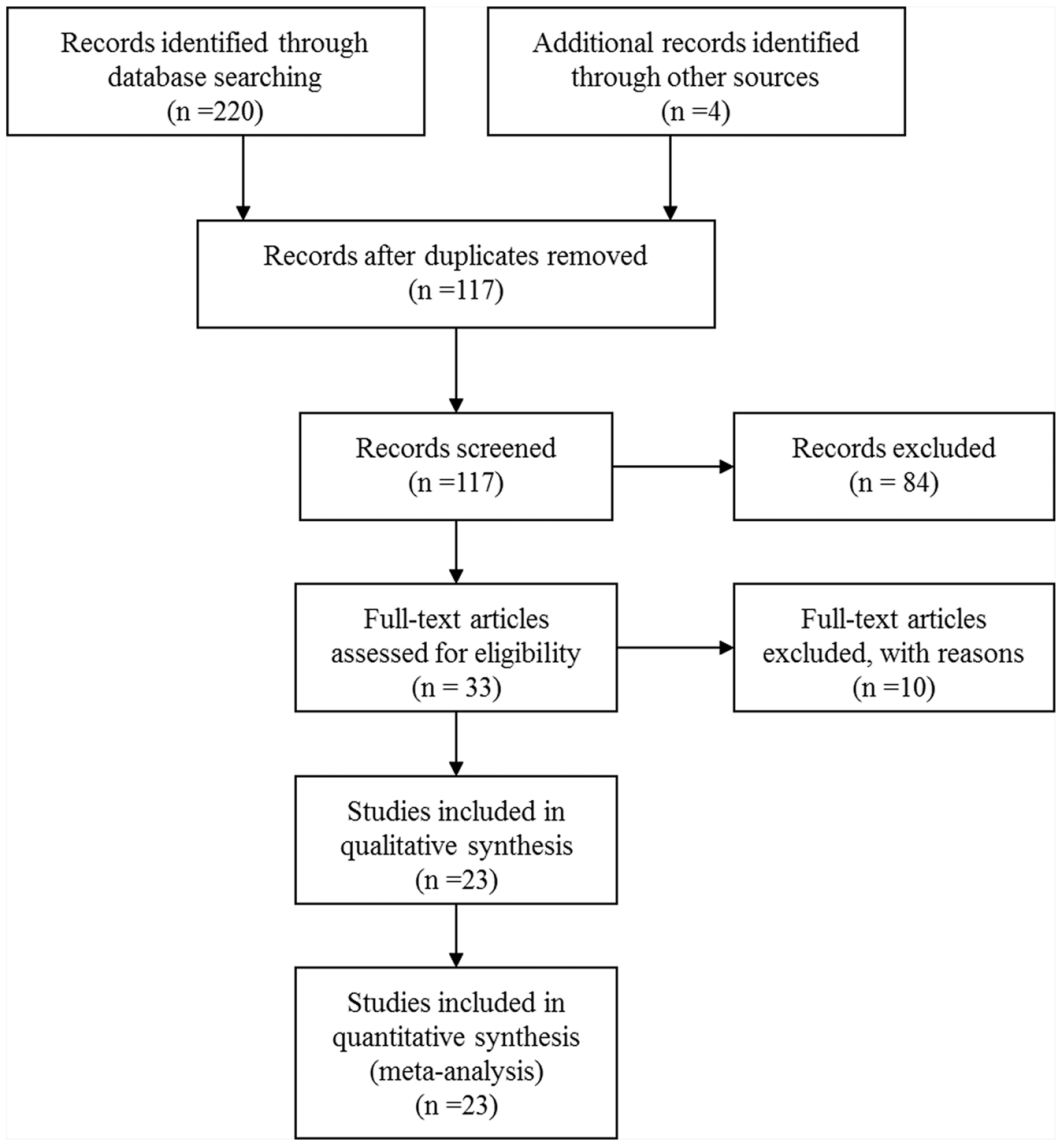
The study search and selection process.

The searching process and selection studies was performed in Figure 1 and the characteristics of the 23 included studies [12], [24], [25], [26], are summarized in Table 1. Among the 32 related epidemiological studies, 2 were excluded because of their examination of other diseases related to T2DM [46], [47]; 4 were removed because of the absence of dichotomous variables, OR or RR, and the CI [48], [49], [50], [51]; 2 were excluded because the biomarkers were *p,p′*-DDT, PCB-126 [52] and PCB-170 [10], and not the ones (*p,p′*-DDE, PCBs, PCB-153) we selected for this study; and 1 was excluded because of the combination of both type 1 and type 2 diabetes [53].

**Table 1:** Study characteristics.

Reference	Country	Study design	age	Exposure pathway	biomarker	Biologic specimen	Cases/controls	OR or RR (95%CI)
Arrebola2013	Spain	Cross-sectional	16-	Background	*p,p′*-DDE	Serum lipid	34/352	1.69(0.54–5.22)^a^
Wu2012	USA	Prospective	30–55	Nurses	PCB-153	Serum lipid	10/214	2.19(0.72–6.68)^a^
			30–55	Nurses	PCB-153	Serum lipid	6/135	0.76(0.22–2.60)^a^
			30–55	Nurses	PCBs	Serum lipid	9/215	1.30(0.43–3.93)^a^
			30–55	Nurses	PCBs	Serum lipid	7/134	0.79(0.23–2.71)^a^
			30–55	Nurses	*p,p′*-DDE	Serum lipid	9/215	1.32(0.41–4.27)^a^
			30–55	Nurses	*p,p′*-DDE	Serum lipid	10/131	1.79(0.54–5.86)^a^
Persky2012	USA	Cross-sectional	35-	Electrical utilities company men	PCB-153	Serum lipid	7/56	3.1(1.2–7.8)^a^
			35-	Electrical utilities company men	PCBs	Serum lipid	7/56	3.0(1.3–7.2)^a^
Gasull2012	Spain	Cross-sectional	18–74	Background	PCB-153	Serum	77/192	1.6(1.2–2.4)^a^
			18–74	Background	PCBs	Serum	77/193	1.7(1.1–2.6)^a^
			18–74	Background	*p,p′*-DDE	Serum	73/207	1.1(0.7–1.7)^a^
Silverstone2012	USA	Cross-sectional	18-	PCBs plant area	PCBs	Serum	78/157	2.78(1.00–7.73)
			18–55	PCBs plant area	PCBs	Serum	27/111	4.78(1.11–20.6)
			55-	PCBs plant area	PCBs	Serum	51/47	4.19(0.26–68.12)
Lee 2011	Sweden	Prospective	70	background	PCB-153	Serum	12/277	1.7(0.5–6.2)
			70	background	*p,p′*-DDE	Serum	16/271	2.1(0.7–6.3)
Airaksinen2011	Finland	Cross-sectional	70	background	PCB-153	Serum lipid	69/398	1.64(0.92–2.93)^b^
			70	background	PCB-153	Serum lipid	10/121	1.03(0.25–4.18)^b^
			70	background	PCB-153	Serum lipid	23/184	1.97(0.75–5.23)^b^
			70	background	PCB-153	Serum lipid	36/93	2.3(0.87–6.11)^b^
			70	background	*p,p′*-DDE	Serum lipid	62/398	1.75(0.96–3.19)^b^
			70	background	*p,p′*-DDE	Serum lipid	7/120	0.88(0.18–4.35)^b^
			70	background	*p,p′*-DDE	Serum lipid	18/175	1.91(0.69–5.27)^b^
			70	background	*p,p′*-DDE	Serum lipid	37/103	1.82(0.71–4.65)^b^
Tanaka 2011	Japan	Cross-sectional	40–64	background	PCB-153	Serum	32/85	0.95(0.90–1.00)^a^
			40–64	background	PCB-153	Serum lipid	32/85	0.73(0.51–1.07)
Grandjean2011	Faroe Islands	Cross-sectional	70–74	Aquatic product	PCBs	Serum lipid	168/544	1.11(0.91–1.35)^a^
			70–74	Aquatic product	*p,p′*-DDE	Serum lipid	168/544	1.01(0.87–1.16)^a^
Son2010	Korea	Cross-sectional	40-	Background	*p,p′*-DDE	Serum	27/26	26.6(2.0–349.1)
			40-	Background	*p,p′*-DDE	Serum lipid	28/26	12.7(1.9–83.7)
Everett2010	USA	Cross-sectional	20-	Background	*p,p′*-DDE	Serum lipid	334/2715	1.08(0.58–2.03)^a^
Ukropec2010	Slovakia	Cross-sectional	21–75	Heavy pollution	PCBs	Serum lipid	120/699	1.86(1.09–3.17)
			21–75	Heavy pollution	*p,p′*-DDE	Serum lipid	125/694	1.94(1.11–3.78)
Lee2010	USA	Perspective	18–30	Background	PCB-153	Serum lipid	39/45	0.8(0.2–2.6)^a^
			18–30	Background	*p,p′*-DDE	Serum lipid	47/45	0.7(0.2–1.9)^a^
Philibert2009	Canada	Cross-sectional	15–86	Aquatic product	PCB-153	Serum	25/101	4.91(1.27–19.01)
			15–86	Aquatic product	PCB-153	Serum lipid	25/101	6.46(2.07–36.63)
			15–86	Aquatic product	PCBs	Serum	25/101	4.91(1.27–19.01)
			15–86	Aquatic product	PCBs	Serum lipid	25/101	5.51(1.26–24.07)
			15–86	Aquatic product	*p,p′*-DDE	Serum	25/101	6.11(1.37–27.3)
			15–86	Aquatic product	*p,p′*-DDE	Serum lipid	25/101	3.56(0.97–13.08)
Rignell-Hydbom2009	Sweden	Prospective	50–59	Background women	PCB-153	Serum	371/371	0.99(0.71–1.4)^c^
			50–59	Background women	PCB-153	Serum	208/208	0.91(0.59–1.4)^c^
			50–59	Background women	PCB-153	Serum	163/163	1.1(0.66–1.9)^c^
			50–59	Background women	PCB-153	Serum	107/107	1.4(0.72–2.6)^c^
			50–59	Background women	PCB-153	Serum	74/74	1.4(0.67–3.1)^c^
			50–59	Background women	PCB-153	Serum	39/39	1.6(0.61–4.0)^c^
			50–59	Background women	*p,p′*-DDE	Serum	371/371	1.1(0.76–1.5)^c^
			50–59	Background women	*p,p′*-DDE	Serum	208/208	0.9(0.57–1.4)^c^
			50–59	Background women	*p,p′*-DDE	Serum	163/163	1.3(0.78–2.2)^c^
			50–59	Background women	*p,p′*-DDE	Serum	107/107	1.5(0.8–2.8)^c^
			50–59	Background women	*p,p′*-DDE	Serum	74/74	2.5(0.97–6.4)^c^
			50–59	Background women	*p,p′*-DDE	Serum	39/39	5.5(1.2–25)^c^
Turyk2009	USA	Prospective	25–76	Aquatic product	*p,p′*-DDE	Serum	24/285	7.1(1.6–31.9)
			25–76	Aquatic product	PCBs	Serum	21/293	1.8(0.6–5.0)
Wang2008	Taiwan, China	Cross-sectional	30-	Rice-bran oil men	PCBs	Serum	155/152	1.7(0.7–4.6)^a^
			30-	Rice-bran oil women	PCBs	Serum	233/218	5.5(2.3–13.4)^a^
Codru2007	USA	Cross-sectional	30-	Background	PCBs	Serum		2.8 (0.7–10.8)^a^
			30-	Background	PCBs	Serum lipid		2.6 (0.8–8.1)^a^
			30-	Background	PCB-153	Serum		3.0 (0.7–12.8)^a^
			30-	Background	PCB-153	Serum lipid		1.4 (0.4–4.8)^a^
			30-	Background	*p,p′*-DDE	Serum		2.6 (0.8–8.8)^a^
			30-	Background	*p,p′*-DDE	Serum lipid		2.4 (0.7–8.3)^a^
Rignell-Hydbom2007	Sweden	Cross-sectional	29–59	Aquatic product women	PCB-153	Serum lipid	15/528	1.4(0.8–2.5)^a^
			29–59	Aquatic product women	*p,p′*-DDE	Serum lipid	15/528	1.3(1.1–1.5)^a^
Cox2007	USA	Cross-sectional	20–74	Background	*p,p′*-DDE	Serum	45/768	2.63(1.2–5.8)^a^
			20–74	Background	*p,p′*-DDE	Serum lipid	35/560	1.5(0.8–2.9)^a^
Lee2006	USA	Cross-sectional	12-	Background	PCB-153	Serum lipid	52/598	6.8(3.0–15.5)
			12-	Background	*p,p′*-DDE	Serum lipid	69/704	4.3(1.8–10.2)
Rylander2005	Sweden	Cross-sectional	49–84	Aquatic product	PCB-153	Serum lipid	22/358	1.16(1.03–1.32)
			49–84	Aquatic product	*p,p′*-DDE	Serum lipid	22/358	1.05(1.01–1.09)
Fierens2003	Belgium	Case-control	50.3–59.4	Background	PCBs	Serum lipid	9/248	7.6(1.58–36.3)

a Different models adjusted by confounding, such as sex, age, BMI, total cholesterol and triglycerides, and various compounds.

b stratified by BMI.

c stratified by the years diagnosed after the baseline investigation.

From the 23 remaining studies, 1 were case-control studies [54], 18 were cross-sectional studies [24], [25], [26], [27], [28], [29], [30], [31], [32], [33], [35], [36], [37], [38], [39], [40], [41], [42], [44], and only 4 study was a prospective study [12], [34], [43], [45]. 10 studies were conducted in the United States [12], [25], [26], [32], [34], [38], [40], [41], [45], 4 in Sweden [30], [36], [39], [42], and the rest were conducted in Japan [27], the Faroe Islands [28], Korea [31], Slovakia [33], Canada [35], Belgium [43], Taiwan, China [37], Spain [24], [44], and Finland [29].

13 studies used PCB-153 [24], [26], [27], [29], [30], [34], [35], [36], [38], [39], [41], [42], [45], 11 studies used PCBs [12], [24], [25], [26], [28], [33], [35], [37], [38], [44], [45] and 18 studies used *p,p′*-DDE as a biomarker [24], [28], [29], [30], [31], [32], [33], [35], [36], [38], [39], [40], [41], [42], [44], [45]. Among these studies, different models were established to assess the OR or RR, and the related CI was adjusted by confounders such as age, body mass index (BMI), sex, or other factors. 2 studies estimated body burden levels of OCPs in both wet weight values (serum sample) and lipid-standardized values (serum lipid sample) [35], [38]. In addition, 2 studies estimated the OR or RR of both men and women [25], [37], and 1 study discussed the groups under and over the age of 55 years and also the total group separately [25]. The risk estimates from Wu et al. were obtained from two independent study [45].

In most studies, the study population was selected by background exposure to OCPs [24], [27], [29], [30], [31], [32], [34], [36], [38], [40], [41], [43], [44], [45]. However, aquatic product exposure [12], [28], [35], [39], [42], heavy pollution area exposure [25], [26], [33], and specific diet exposure, such as rice-bran oil exposure [37], were also estimated in some studies. As a susceptible population, women were selected [36], [39], [45] in 3 studies. 6 studies collected serum as biologic specimens [12], [24], [25], [30], [36], [37], while 12 chose serum lipids [26], [28], [29], [32], [33], [34], [39], [41], [42], [43], [44], [45]. 5 studies detected the pollutants in both serum and serum lipid specimens [27], [31], [35], [38], [40].

### Quality Assessment

The quality factor scores for the 23 studies are listed (Table S2). From the results of the quality assessment, all the included epidemiological studies accorded with most of the quality criteria we listed, but the items of participation rate, blind laboratory testing, data dredging, specific exposure measurement, and adequate adjustment for confounding were different among the original studies. The total quality scores were in the range from 9 to 14 with a possible maximum score of 16, reflecting the existing of study design limitations. More recent studies tended to have higher quality scores. Among these studies, only 4 studies had the external validity that participation rate for cases and controls reaches 70% [34], [36], [40], [43]. Only 3 studies reported having made an attempt to blind those measuring the main outcomes of the OCPs exposure [29], [31], [37]. Most of the included studies got the scores of other 11 items.

### Main Analysis

**Figure 2:**
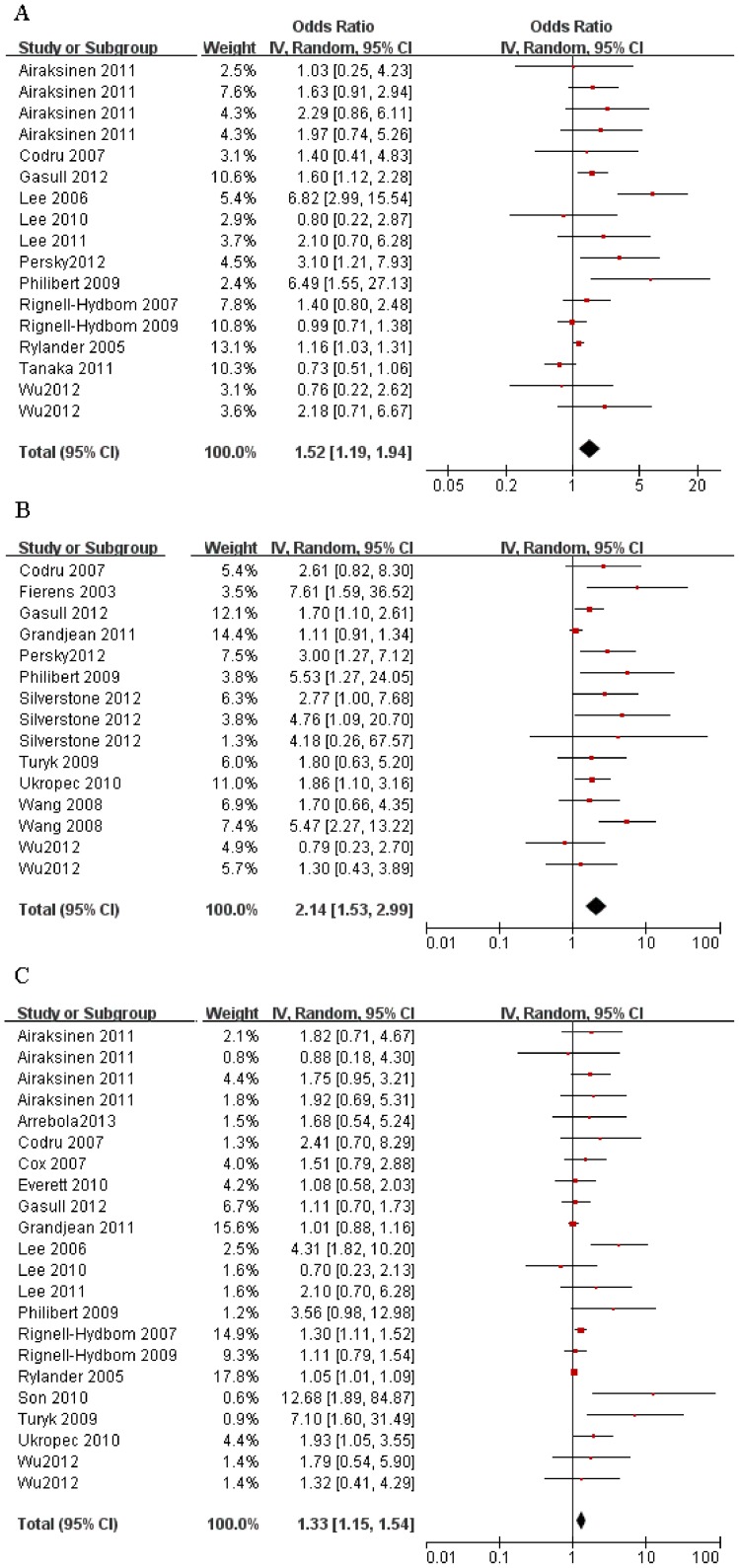
Subgroup analysis forest plots of the studies on T2DM risk from exposure to all three biomarkers. (*A*) Result of exposure to PCB-153. (*B*) Result of exposure to PCBs. (*C*) Result of exposure to *p,p′*-DDE.

23 studies, contributing a total of 73 OR or RR estimators met the inclusion criteria and were taken into consideration. When combining the main data of all studies, the exposure to all 3 biomarkers showed positive associations with the prevalence of T2DM. The combined OR estimate of PCB-153 was 1.52 (95% CI, 1.19–1.94), for PCBs was 2.14 (95% CI, 1.53–2.99), and for *p,p′*-DDE was 1.33 (95% CI, 1.15–1.54) based on a random-effects model. Forest plots of the 3 organochlorine biomarkers, which show the weight of each study and the combined OR estimates, are provided in Figure 2. Considering the high evidence of heterogeneity for PCB-153 (*I* ^2^ = 64%), PCBs (*I* ^2^ = 59%) and *p,p′*-DDE (*I* ^2^ = 56%), subgroup meta-analyses were conducted for the OR combining and further analyses of sources of heterogeneity. The results for the meta-analyses of 3 organochlorine biomarkers (PCB-153, PCBs, and *p,p′*-DDE) and their subgroups were analyzed and are summarized in Table 2.

**Table 2:** Subgroup analysis of the included epidemiological studies.

Subgroup	NO. of studies	Weight	Summary OR (95%CI)	Q test (p)	*I^2^*
PCB-153					
Background exposure	10	76.6%	1.57(1.13–2.19)	0.0004	65%
Specific exposure	3	23.4%	-	0.05	66%
Total	13	100%	1.52(1.19–1.94)	0.0002	64%
Perspective study	4	23.5%	1.05(0.78–1.40)	0.60	0%
Cross-sectional study	9	76.5%	1.69(1.24–2.31)	<0.0001	72%
Total	13	100%	1.50(1.18–1.92)	0.0002	63%
General population	10	74.7%	1.69(1.23–2.33)	<0.0001	71%
Women	3	25.3%	1.11(0.85–1.46)	0.41	0%
Total	13	100%	1.52(1.19–1.94)	0.0002	64%
Serum	6	38.8%	1.36(0.95–1.95)	0.002	74%
Serum lipid	10	61.2%	1.62(1.18–2.24)	0.0002	67%
Total	13	100%	1.44(1.18–1.76)	<0.00001	74%
PCBs					
Background exposure	4	31.6%	1.74(1.22–2.48)^a^	0.22	30%
Specific exposure	7	68.4%	2.39(1.52–3.77)	0.001	67%
Total	11	100%	2.14(1.53–2.99)	0.002	59%
Case-control study	2	10.9%	2.83(1.18–6.81)^a^	0.14	55%
Cross-sectional study	8	89.1%	2.28(1.55–3.34)	0.001	66%
Total	10	100%	2.36(1.64–3.41)	0.0008	64%
General population	9	80.4%	2.18(1.50–3.16)	0.006	59%
Women	2	19.6%	1.88(0.56–6.26)	0.02	74%
Total	11	100%	2.19(1.54–3.13)	0.001	62%
Serum	6	48.3%	2.31(1.71–3.11)^a^	0.36	8%
Serum lipid	7	51.7%	1.91(1.20–3.04)	0.009	62%
Total	11	100%	2.24(1.62–3.10)	0.001	58%
p,p′-DDE					
Background exposure	12	45.2%	1.39(1.16–1.67)^a^	0.18	25%
Specific exposure	6	54.8%	1.20(1.01–1.43)	0.001	75%
Total	18	100%	1.33(1.15–1.54)	0.0007	56%
Perspective study	4	14.7%	1.16(0.87–1.55)^a^	0.64	0%
Cross-sectional study	13	85.3%	1.33(1.13–1.56)	0.0006	62%
Total	17	100%	1.29(1.12–1.48)	0.003	52%
General population	15	72.9%	1.41(1.17–1.71)	0.0008	59%
Women	3	27.1%	1.27(1.10–1.46)^a^	0.79	0%
Total	18	100%	1.33(1.15–1.54)	0.0007	56%
Serum	8	24.4%	2.22(1.32–3.73)	0.005	66%
Serum lipid	14	75.6%	1.34(1.14–1.57)	0.001	58%
Total	18	100%	1.45(1.24–1.70)	<0.0001	62%

a Based on fixed model, others based on random model.

### Subgroup and Sensitivity Analyses

From the characteristics summary of the epidemiological studies, exposure levels (background or high concentration exposure), study design (case-control or cross-sectional study), population selection (general population or women), and biologic specimen (serum or serum lipid) were chosen as the stratifications for subgroup analyses to find the sources of heterogeneity.

For PCB-153, the exposure subgroup analyses may not be a heterogeneity source from the increased results of *I* ^2^ test. When the studies were stratified by the study design, heterogeneity and inconsistency among the epidemiological studies were eliminated in the perspective subgroup (*I* ^2^ = 0%). When the studies were divided by the sex of the population, the consistency was observed among the women subgroup (*I* ^2^ = 0%) and the heterogeneity between studies remained high for general population group (*I* ^2^ = 71%). Finally, when the studies were stratified by biologic specimen, the high inconsistencies in both the serum (*I* ^2^ = 74%) and serum lipid subgroup (*I* ^2^ = 67%) also existed.

For PCBs, the background exposure subgroup (*I* ^2^ = 30%) the subgroup of serum specimen exposure (*I* ^2^ = 8%) resulted in a statistically decreasing heterogeneities from the total studies (*I* ^2^ = 64%). The subgroup analysis of the case-control subgroup (*I* ^2^ = 55%, n = 2 only), general population (*I* ^2^ = 59%) and women population (*I* ^2^ = 74%) gave the result that they still had relatively high heterogeneity.

The subgroup of background exposure from *p,p′*-DDE showed decreased heterogeneity (*I* ^2^ = 25%) compared with total studies (*I* ^2^ = 56%), and a risk factor was found from the combined OR (OR, 1.49; 95% CI, 1.18–1.88). Decreased heterogeneity was also found in the perspective group (*I* ^2^ = 0%) and women subgroup (*I* ^2^ = 0%) with a combined OR of 1.16 (95% CI, 0.87–1.55) and 1.27 (95% CI, 1.10–1.46). The heterogeneities in subgroups classified by biologic specimen were still in relatively high levels.

In general, findings from each sensitivity analysis did not substantially alter the results of the overall pooled estimate OR using the random effects model in direction and magnitude. Exclusion of the studies with the highest and lowest percentage weight, the highest and lowest quality scores, and the lowest or highest estimator of OR performed consistently with the pooled estimator OR for all indicators, including PCB-153, PCBs, and *p,p′*-DDE (data not shown).

### Publication Bias

**Figure 3:**
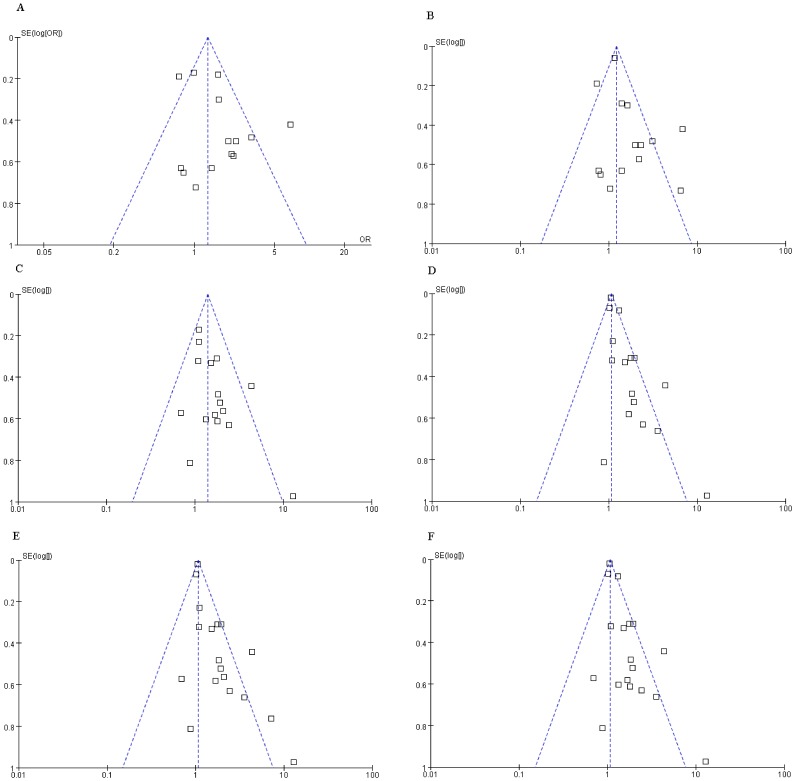
A funnel plot of SE versus ln(OR) for the meta-analyses. (*A*) Funnel plot for the meta-analysis on T2DM from background exposure to PCB-153. (*B*) Funnel plot for the subgroup analysis of serum lipid on T2DM from exposure to PCB-153. (*C*) Funnel plot for the subgroup analysis on T2DM from background exposure to *p,p′*-DDE. (*D*) Funnel plot for the subgroup analysis of the cross-sectional study on T2DM from exposure to *p,p′*-DDE. (*E*) Funnel plot for the subgroup analysis of the general population on T2DM from exposure to *p,p′*-DDE. (*F*) Funnel plot for the subgroup analysis of serum lipid on T2DM from exposure to *p,p′*-DDE.

A funnel plot of standard error (SE) versus ln(OR) for the meta-analyses of the relationships between OCPs and T2DM, in which the number of studies was more than 10, are presented in Figure 3. Visual inspection of the funnel plot suggests that risk estimates stemmed mostly from large, precise studies, which are distributed in the superior part of the figure; however, possible publication bias was found from the evidence of asymmetry of some subgroup meta-analyses. Additionally, exclusion of studies published with non-English and other factors such as differences in study quality or heterogeneity, sample size, and study design may be other reasons for the asymmetries of the funnel plots.

## Discussion

While a large number of studies about the association between OCPs exposure and the prevalence of T2DM were published, only few cohort studies are available. Among these studies, inconsistency was found among most of these epidemiological studies in different populations and different sources of exposure. However, our combined estimates of meta-analyses demonstrated a modest but statistically significant increase in the odds of T2DM with exposure to OCPs. For instance, a 52% increase of T2DM resulted from an exposure to PCB-153. In addition, all subgroup analyses stratified by exposure levels, design of the studies, study subjects and biologic specimen resulted in positive correlations. The consistency in the magnitude of increased risk indicates that this is unlikely to be a chance finding and these increased risks support the suggestion that exposure to OCPs may be a potential causal factor for prevalence of T2DM.

To determine the sources of heterogeneity of the studies and obtain the pooled estimates of PCBs, PCB-153, and *p,p′*-DDE in subgroups, subgroup analysis was conducted by stratified exposure levels, study designs, study subjects and biologic specimens. Specific exposure, such as seafood consumption and living in a high exposure area, did not show an increased risk of T2DM compared with background exposure studies. This result may be attributed to the limited studies of specific exposure and the discrepant results of exposure concentration in serum levels such as 70 to 70000 ng/g and 44.33 to 8863 ng/g PCBs found in a serum lipid in two aquatic food consumption studies [28], [35], and 148 to 101413 ng/g PCBs found in a study on serum lipid in a heavily polluted area [33]. Additionally, the heterogeneity analysis indicated that background exposure was a group with smaller heterogeneity and specific exposure with higher heterogeneity for the significant differences of the exposure. With regard to study design and subjects, from the consistent *I* ^2^ test results of the subgroup analyses and the limited studies of the perspective study subgroup and women's subgroup, it may not be an obvious heterogeneity source of the meta-analyses for all organochlorine biomarkers in this study. The biologic specimen was considered a heterogeneity source for the decreasing *I* ^2^ test results from the serum subgroup.

Some studies preferred express OCP concentrations per weight of lipid rather than on a whole weight for the lipophilic character of the pollutants. Total lipid was defined using different formulas as total lipids (mg/dL) = 2.27×total cholesterol (mg/dL)+triglycerides (mg/dL)+62.3 [31], [38] and total lipids = 1.13+1.13×(cholesterol+ triglycerides) [39]. Various definitions of total lipids may be one of the reasons that serum lipid is an obviously heterogeneity source. The gravimetric analysis of cholesterol and triglycerides with different detected method is another labile factor [55]. While, OCPs is lipophilic and likely to concentrate in serum lipid, so many studies used the concentrations based on serum lipid to present the residual levels in humans.

Furthermore, the different exposure contrast used in each study population may also be a larger source of heterogeneity. For instance, some studies [24], [33] evaluated the increase in the odds comparing the 80th and the 20th percentiles of OCP concentrations, some studies used the quartiles for the comparison [25], [34], while Airaksinen et al. calculated OR on the basis of percentile intervals <10th, 10th to <50th, 50th to <90th, and ≥90th. The diverse categories may cause the great heterogeneity among studies [29].

The original studies may be subject to limitations mainly related to the quality of potential sources of bias, exposure assessment, confounding, and the validity of the enrolled data. The bias in internal validity is most likely attributed to the misclassification of diabetes outcomes that only rely on self-reporting the prevalence of T2DM, the use of oral antidiabetic drugs or insulin, or that patients were on a specific diet [25], [30], [31], [34], [37], [38], [42], [43] but lack of an accurate fasting glucose. Publication bias was also among the potential limitations from the evidence of asymmetry of the funnel plot. Some of the studies included in the meta-analysis were based on the same data, meaning that data for some subjects were included twice. For instance, some subjects from NHANES 1999–2004 was used both in the study of Everett et al. [32] and Lee et al. [41] and in both two studies, *p,p′*-DDE concentrations in serum were used as the indicator of the OCPs exposure. This may be a reason of the publication bias. However, this was not enough to negate the overall conclusion of an increased risk based on limited evidence for the deficit in small negative studies with effect sizes smaller than those from larger studies and in non-English published original studies.

It has been argued that exposure measurement is a typical effect factor for the quality of environmental epidemiology studies [56]. Though most studies gave a sufficient exposure gradient in the T2DM risk assessment (a dose-dependent manner across quantiles of the exposure levels), multiple quantile categories, such as tertile [31], [44], [45], quartile [24], quintile [25], percentile intervals [29] were used for the OR estimate via setting the lowest quantile or percentile intervals as the reference. Another limitation is the unspecific exposure measurements. In the present meta-analysis, we selected representative PCB-153, PCBs, and *p,p′*-DDE as the biomarkers. For PCBs, 209 congeners existed in the environment, and the selection of the representatives PCBs varied among studies. For instance, 15 PCB congeners were selected in a study on high level exposure [33], while another study conducted in a fish consumption group selected only 8 PCB congeners as representatives of PCBs [35].

Confounding is also a potentially limited factor. Relevant confounders were selected from different adjusted models including basic demographics such as age, sex, and BMI and other major covariates, such as serum lipids, serum triglycerides, total cholesterol, fish consumption, smoking and alcohol. Models that were adjusted differently, including crude estimates, models with basic demographic variables, and models with all major confounders, led to discrepancies in the estimated OR. In the present meta-analyses, we selected the models with the most confounders which may give more accurate effects values to create pooled OR estimates. For instance, in a marine food consumption study, the model with basic demographic and all of the major confounders, was set and adjusted to evaluate the OR estimates [28].

Other than bias, exposure measurement, and confounding, the effect of the validity of the enrolled data is important. When outcome of interest was rare, such as the prevalence of cancer or birth defect, one can generally ignore the distinctions among the various measures of relative risk [57]. According to statistical data from the Ministry of Health of The People's Republic of China, the prevalence of T2DM were about 5 times the prevalence of cancer. In this study, we combined the binary variables of OR and RR. Considering the high prevalence of T2DM, the data processing may impact the consequence of the meta-analysis.

From the systematic screening of the relevant studies about the prevalence of T2DM exposure to OCPs, we found several other systematic reviews [58], [59], [60]. However, this study was the first meta-analysis to our knowledge to evaluate the pooled effect values. A previous systematic review assessed the risk for developing T2DM from exposure to organochlorine pesticides [58] but only analyzed the results at the qualitative and quantitative levels. Although the positive relationships were the same as our results, many limitations and uncertainties were proposed in that review. Exposure to OCPs cannot be concluded as being the only contributor to the prevalence of T2DM, and many factors other than exposure to OCPs may be causative for T2DM, such as obesity, race, gender, age, genetic susceptibility, dietary habits and lifestyle. From another review that discussed the impacts of OCPs on metabolic health [60], no associations between OCPs exposures and stages of glucose intolerance or markers of insulin resistance were observed [61]. This specific result is not in our meta-analysis because of the misclassification of T2DM outcomes, which may cause bias in our study. Additionally, a cross-sectional study conducted in Swedish [51] found that none of the PCB congeners selected were significantly associated with diabetes in age, BMI, weight change and region adjusted analyses. However, because of the lack of OR or RR estimates, we removed this study in our meta-analysis. Another cross-sectional study of 380 Swedish fishermen and their wives found significantly increased risk from exposure to PCB-153 congener in men but not in women [42]. For the subjects were either women or all overall population, it was not included. In the review of the relationship of PCBs with T2DM and hypertension, the author attributed these two results to hypothesis generating [59].

Overall, the findings from the present meta-analysis provide quantitative evidence consistent with the hypothesis that exposure to OCPs is a contributing risk factor for the prevalence of T2DM. From the heterogeneity analysis, the specific exposure and biologic specimen of serum lipid may be the heterogeneity sources for the large disparities of the concentration of this class of environmental pollutants. Based on our sensitivity analysis, sources of bias, exposure assessment, and confounding are unlikely to significantly affect the results. In regards to the possible observed publication bias, more studies with small samples and adverse results should be included in future research. Apart from the conventional etiologies that include genetic susceptibility, metabolic disorder and obesity, the finding of the meta-analysis indicates that environmental factors, especially exposure to OCPs, may also be a risk factor of T2DM.

## Supporting Information

Table S1 **Modified Downs and Black checklist for the quality assessment of epidemiological studies.**
(DOC)

Table S2 **Quality assessment of the included epidemiological studies.**
(DOC)

Checklist S1 **PRISMA (Preferred Reporting Items for Systematic Reviews and Meta-Analyses) 2009 Checklist.**
(DOC)
