# Supplementary material for: Exposure to Organochlorine Pollutants and Type 2 Diabetes: A Systematic Review and Meta-Analysis
Source: PLoS One. 2014 Oct 15;9(10):e85556. doi: 10.1371/journal.pone.0085556 (PMC4198076; doi:10.1371/journal.pone.0085556)
Supplement: Table S1 — Modified Downs and Black checklist for the quality assessment of epidemiological studies. (DOC) [file pone.0085556.s001.doc]

**Table S1. Modified Downs and Black checklist for the quality assessment of epidemiological studies**

| Factor | Score |
| --- | --- |
| External validity  1. Were the subjects asked to participate in the study representative of the entire population from which they were recruited? |  |
|  | 1 |
| 2. Were those subjects who were prepared to participate representative of the entire population from which they were recruited? Participation rate for cases and controls of at least 70% | 1 |
| Subtotal | 2 |
| Internal Validity-Bias  3. Was an attempt made to blind those measuring the main outcomes of the exposure? |  |
|  | 1 |
| 4. If any of the results of the study were based on “data dredging”, was this made clear? | 1 |
| 5. Were the statistical tests used to assess the main outcomes appropriate? | 1 |
| 6. Was compliance with the intervention/s reliable? | 1 |
| 7. Were the main outcome measures used accurate (valid and reliable)? | 1 |
| Subtotal | 5 |
| Internal validity-exposure measurement  8．Were measures of exposure robust? Exposure status was either documented or determined via biomarker (2); used small area ecological measures, job titles, or was self-reported (1); was based on large area ecological measures (0). |  |
|  | 2 |
| 9．Was there a sufficient exposure gradient? The degree of variability between categories of exposure level was certain or not. | 1 |
| 10．Were measures of exposure specific? Exposure measures were specific (2); based on broader, chemically-related groups (1); based on broad groupings of diverse chemical and toxicological properties (0). | 2 |
| Subtotal | 5 |
| Internal Validity – Confounding  11. Were the cases and controls recruited from the same population? |  |
|  | 1 |
| 12. Were the cases and controls recruited over the same period of time? | 1 |
| 13. Was there adequate adjustment for confounding in the analyses from which the main findings were drawn? The study collected data on all major (2), some (including basic demographic only) (1), or no (0) potential confounders and assessed their effect in analysis. | 2 |
| Subtotal | 4 |
| Total | 16 |
