# Supplementary material for: Exposure to Organochlorine Pollutants and Type 2 Diabetes: A Systematic Review and Meta-Analysis
Source: PLoS One. 2014 Oct 15;9(10):e85556. doi: 10.1371/journal.pone.0085556 (PMC4198076; doi:10.1371/journal.pone.0085556)
Supplement: Table S2 — Quality assessment of the included epidemiological studies. (DOC) [file pone.0085556.s002.doc]

Table S2. Quality assessment of the included epidemiological studies

| Reference | External validity | | Internal validity | | | | | | | | | | | Total |
| --- | --- | --- | --- | --- | --- | --- | --- | --- | --- | --- | --- | --- | --- | --- |
|  |  | | Bias | | | | | Exposure measurement | | | Confounding | | | score |
|  | Item1 | Item2 | Item3 | Item4 | Item5 | Item6 | Item7 | Item8 | Item9 | Item10 | Item11 | Item12 | Item13 | 16 |
| Gasull 2012 | 1 | 0 | 0 | 0 | 1 | 1 | 1 | 2 | 1 | 2 | 1 | 1 | 2 | 13 |
| Silverstone 2012 | 1 | 0 | 0 | 0 | 1 | 1 | 0 | 2 | 1 | 1 | 1 | 1 | 2 | 11 |
| Tanaka 2011 | 1 | 0 | 0 | 0 | 1 | 1 | 1 | 2 | 0 | 2 | 1 | 1 | 2 | 12 |
| Grandjean 2011 | 1 | 0 | 0 | 1 | 1 | 1 | 1 | 2 | 1 | 1 | 1 | 1 | 2 | 13 |
| Airaksimen 2011 | 1 | 0 | 1 | 1 | 1 | 1 | 1 | 2 | 1 | 2 | 1 | 1 | 1 | 14 |
| Lee 2011 | 1 | 0 | 0 | 1 | 1 | 1 | 0 | 2 | 1 | 2 | 1 | 1 | 2 | 13 |
| Son 2010 | 1 | 0 | 1 | 1 | 1 | 1 | 0 | 2 | 1 | 2 | 1 | 1 | 2 | 14 |
| Everett 2010 | 1 | 0 | 0 | 1 | 1 | 1 | 1 | 2 | 1 | 2 | 1 | 1 | 2 | 14 |
| Ukropec 2010 | 1 | 0 | 0 | 1 | 1 | 0 | 1 | 2 | 1 | 1 | 1 | 1 | 1 | 11 |
| Lee 2010 | 1 | 1 | 0 | 0 | 1 | 1 | 0 | 2 | 1 | 2 | 0 | 0 | 2 | 11 |
| Philibert 2009 | 1 | 0 | 0 | 1 | 1 | 1 | 1 | 2 | 1 | 2 | 1 | 1 | 0 | 12 |
| Rignell-Hydbom 2009 | 1 | 1 | 0 | 1 | 1 | 1 | 1 | 2 | 1 | 2 | 0 | 1 | 0 | 12 |
| Turyk 2009 | 1 | 0 | 0 | 0 | 1 | 1 | 1 | 2 | 1 | 1 | 0 | 0 | 1 | 9 |
| Wang 2008 | 1 | 0 | 1 | 1 | 1 | 0 | 0 | 2 | 1 | 1 | 1 | 1 | 1 | 11 |
| Codru 2007 | 1 | 0 | 0 | 1 | 1 | 1 | 0 | 2 | 1 | 1 | 1 | 1 | 2 | 12 |
| Rignell-Hydbom 2007 | 1 | 0 | 0 | 1 | 1 | 1 | 1 | 2 | 1 | 2 | 1 | 1 | 1 | 13 |
| Cox 2007 | 1 | 1 | 0 | 1 | 1 | 1 | 1 | 2 | 1 | 2 | 1 | 1 | 1 | 14 |
| Lee 2006 | 1 | 0 | 0 | 1 | 1 | 1 | 1 | 2 | 1 | 1 | 1 | 1 | 2 | 13 |
| Rylander 2005 | 1 | 0 | 0 | 0 | 1 | 1 | 0 | 2 | 1 | 2 | 1 | 1 | 1 | 11 |
| Fierens 2003 | 1 | 1 | 0 | 0 | 1 | 0 | 0 | 2 | 1 | 1 | 1 | 1 | 2 | 11 |
